# Platelet-Derived Growth Factor CC-Mediated Neuroprotection against HIV Tat Involves TRPC-Mediated Inactivation of GSK 3beta

**DOI:** 10.1371/journal.pone.0047572

**Published:** 2012-10-15

**Authors:** Fuwang Peng, Honghong Yao, Halis Kaan Akturk, Shilpa Buch

**Affiliations:** 1 Department of Pharmacology and Experimental Neuroscience, University of Nebraska Medical Center, Omaha, Nebraska, United States of America; 2 Department of Medicine, Creighton Medical Center, Omaha, Nebraska, United States of America; University of Pecs Medical School, Hungary

## Abstract

Platelet-derived growth factor-CC (PDGF-CC) is the third member of the PDGF family, and has been implicated both in embryogenesis and development of the CNS. The biological function of this isoform however, remains largely unexplored in the context of HIV-associated dementia (HAD). In the present study, we demonstrate that exposure of human neuroblastoma cells SH-SY5Y to HIV transactivator protein Tat resulted in decreased intrinsic expression of PDGF-CC as evidenced by RT-PCR and western blot assays. Reciprocally, pretreatment of SH-SY5Y cells with PDGF-CC abrogated Tat-mediated neurotoxicity by mitigating apoptosis and neurite & MAP-2 loss. Using pharmacological and loss of function approaches we identified the role of phosphatidylinositol 3-kinase (PI3K)/Akt signaling in PDGF-CC-mediated neuroprotection. We report herein a novel role about the involvement of transient receptor potential canonical (TRPC) channel 1 in modulation of calcium transients in PDGF-CC-mediated neuroprotection. Furthermore we also demonstrated PDGF-CC-mediated inactivation of the downstream mediator - glycogen synthase kinase 3β (GSK3β) evidenced by its phosphorylation at Ser-9. This was further validated by gain and loss of function studies using cells transfected with either the wild type or mutant GSK3β constructs. Intriguingly, pretreatment of cells with either the PI3K inhibitor or TRPC blocker resulted in failure of PDGF-CC to inactivate GSK3β, thereby suggesting the intersection of PI3K and TRPC signaling at GSK3β. Taken together our findings lead to the suggestion that PDGF-CC could be developed as a therapeutic target to reverse Tat-mediated neurotoxicity with implications for HAD.

## Introduction

Worldwide there are around 40 million people infected with human immunodeficiency virus (HIV). In the late phase of HIV-1 infection, a subset of patients will go on to develop end-organ diseases including HIV-associated dementia (HAD) [1], [2]. Clinically, the disease is characterized by cognitive impairment that is later accompanied by motor symptoms such as gait disturbance and tremor [3]. Pathological manifestation of the syndrome is accompanied by prominent microglial activation, formation of microglial nodules, perivascular accumulations of mononuclear cells, presence of virus-infected multinucleated giant cells, and neuronal damage & loss [4]–[6]. The mechanism(s) underlying the pathogenesis of HAD are complex. Multiple pathways have been implicated in the HIV-mediated neuronal apoptosis/death, including cellular and viral factors.

Although neuronal cell death is a common feature of HIV neuropathogenesis, neurons are rarely infected by HIV-1. It is speculated that cellular and viral toxic products that are released from virus-infected and/or activated cells could be indirectly contributing to neuronal apoptosis [7], [8]. One of the potent viral toxins implicated in neuronal injury/death is the virus transactivator protein, Tat that can both be secreted from infected cells and can also be taken up by the neighboring non-infected cells, including neurons [8], [9]. Tat, a mediator of virus replication, was first identified as a neurotoxin by Nath *et. al* [10]. It was shown to be released by HIV-infected cells and was detected in the cerebrospinal fluid and sera of HIV-infected patients [11], [12]. Previous studies have also demonstrated that Tat can interact with the lipoprotein related protein receptor and be taken up by the neurons, resulting in a series of cytoplasmic and nuclear events [9], [13]–[15].

In the CNS, neuronal homeostasis is a fine balance between neurotrophic versus neurotoxic factors. Various neurotrophic factors have been implicated in the protection of neurons against neurotoxins, such as brain-derived neurotrophic factor (BDNF), nerve growth factor (NGF), glia cell line-derived neurotrophic factor (GDNF) and fibroblast growth factor (FGF) [16]–[19]. Belonging to these family of growth factors is yet another factor, platelet-derived growth factor (PDGF), that is widely expressed in both embryonic and adult CNS, where it is known to exert neurotrophic effects [20]. In the present study, we explored the role of one subtype of PDGF, PDGF-CC that is the third membrane of family of five dimeric ligands (PDGF-AA, BB, CC, DD and AB) [21], [22]. PDGF-CC plays a critical role in embryonic development since its absence during development has been shown to result in postnatal lethality [23]. Similar to other membranes of PDGF family, PDGF-CC is also found distributed in different neuronal tissues, including the brain [24]. The cognate PDGF receptors, -α & -β belong to the family of tyrosine kinases and are widely expressed in the brain. Activation of these receptors by PDGF-CC has been implicated in neuroprotection following ischemic events in the rat [25]. While it is clear that PDGF-CC plays a critical role in the physiological functioning of neurons, its function in Tat-mediated toxicity remain less clear.

Phosphatidylinositol-3 kinase (PI-3 kinase) has been implicated as an important signaling pathway for neuronal survival initiated by various neurotrophic factors [26]–[28]. Although all the effectors downstream of PI-3 kinase that mediate neuronal survival have not been completely identified, protein kinase Akt/glycogen synthase kinase-3β (GSK3β) has been implicated as playing a key role in this process [29], [30]. More recently, signaling via the transient receptor potential (TRP) channels has also been demonstrated to be essential for neuron survival [31], [32]. TRPC channels belong to the family of Ca^2+^-permeable nonselective cation channels that are activated by receptor tyrosine kinases [31], [32]. In the present study we demonstrate the involvement of TRPC and Akt in PDGF-CC mediated neuroprotection against toxicity induced by Tat. Taken together these findings implicate that PDGF-CC could be developed as a therapeutic agent for mitigating Tat-mediated neurotoxicity in HAD.

## Materials and Methods

### Materials

Human neuroblastoma cells (SH-SY5Y) were purchased from American Type Culture Collection (Manassas, VA). The rationale for choosing these cells was based on their ability to mimic the pathways involved in the neurodegenerative processes observed in HIVE [33], [34]. Human recombinant PDGF-CC was purchased from Abcam (Cambridge, MA) and recombinant viral Tat protein (derived from HIV-1 clade B, 1–72 amino acid) was obtained from Kentucky University. Details of Tat production and purification have been previously published [35]–[37].

**Figure 1:**
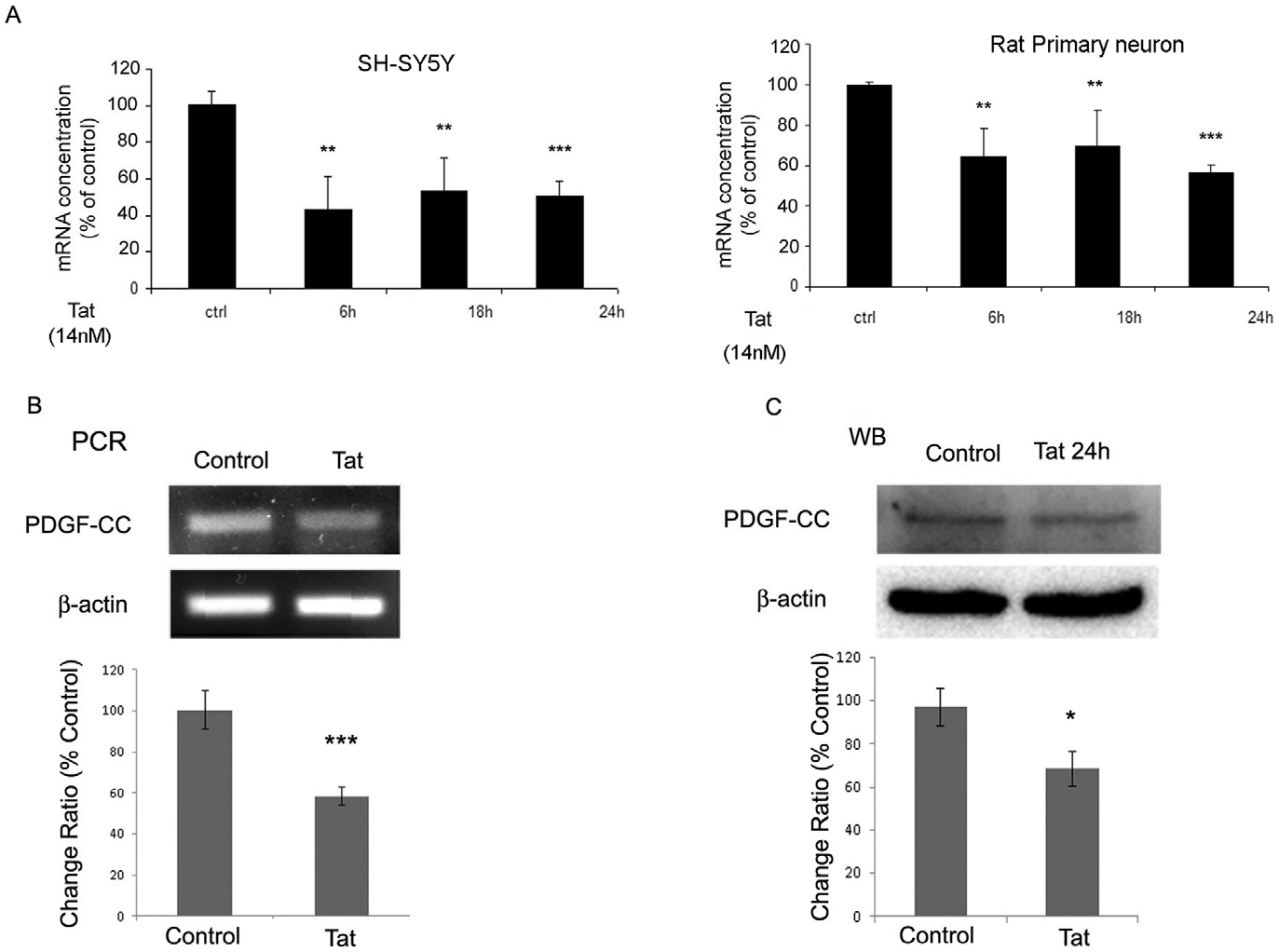
Down-regulation of PDGF-CC in neurons exposed to Tat. SH-SY5Y cells or rat primary neurons were exposed to 14 nM Tat for the indicated times, followed by RNA isolation and assessment of PDGF-CC using real-time PCR (**A**), RT-PCR (**B**), or western blot (**C**). In panels B and C, SH-SY5Y cells were treated for 24 h and monitored for PDGF-CC RNA and protein. Figure shown is a representative of three independent experiments. All data in these figures are presented as mean ± SEM of three individual experiments. *p<0.05, **p<0.01, ***p<0.001 vs control.

### Cell Culture and Treatments

SH-SY5Y cells were plated at a density of 1×10^5^/ml and cultured in Dulbecco’s Modified Eagle Medium: Nutrient Mixture F-12 (DMEM/F12) (Gibco, Gaithersburg, MD) supplemented with heat-inactivated fetal bovine serum (10% v/v) at 37°C in 5% CO_2_. Confluent cells were re-plated at a density of 1–5×10^5^cells/ml for different experiments and differentiated by treatment with 10 µM retinoic acid (Sigma-Aldrich, St. Louis, MO) for 7 days with media change every 2 days. For all of the experiments, cells were serum-starved for 24 h in the presence of 10 µM retinoic acid prior to treatment with PDGF-CC (50 ng/ml; predetermined dose [38]) for 30 min, followed by addition of Tat protein (14 nM). In the experiments involving pharmacological inhibitors, SH-SY5Y cells were pretreated with respective inhibitors (STI-571, EGTA, SKF96365, 2APB, U73122, Calbiochem, San Diego, CA; PI-103, Tocris Bioscience, Ellisville, MO) for 1 h followed by treatment with PDGF-CC and/or Tat as described above.

**Figure 2:**
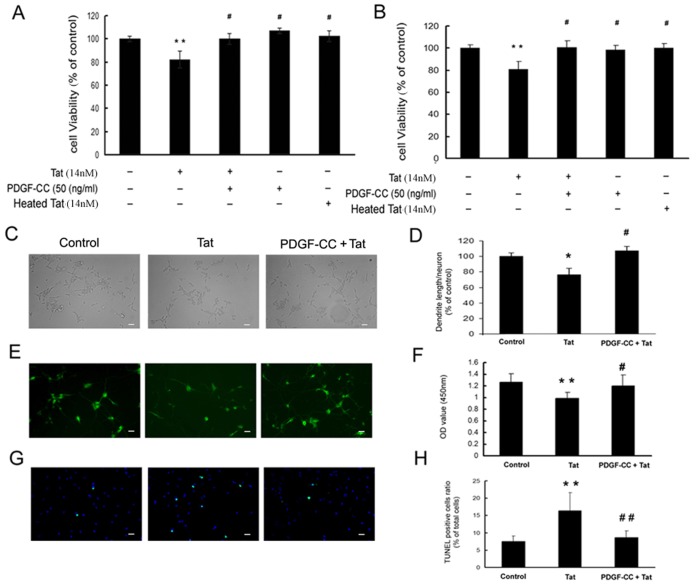
PDGF-CC exerts neuroprotection against Tat toxicity. SH-SY5Y cells (**A**) or Rat primary cortical neurons (**B**) were exposed to Tat (14 nM) or heat-inactivated Tat (14 nM) with or without pretreatment with PDGF-CC (50 ng/ml), and were then subjected to MTT assay. (**C**) SH-SY5Y cells were treated with Tat in presence or absence of PDGF-CC for 5 days and examined phase-contrast microscopy. (**D**) Densitometric scan of neuritis from panel C expressed as a ratio of neurite length/neuron. (**E**) Immunostaining of SH-SY5Y cells treated with PDGF-CC and/or Tat for 3 days with anti-MAP-2 antibody. (**F**) MAP-2 ELISA was done on SH-SY5Y cells treated as described in panel E. (**G**) SH-SY5Y cells were treated with 14 nM Tat and/or 50 ng/ml PDGF-CC for 24 h and were then subjected to TUNEL assay. The data (**H**) were expressed as ratio of TUNEL positive to total cell number in the same view area. For all images, scale bars represent 20 µm. Figure is a representative of three independent experiments. All data in these figures are presented as mean ± SEM of three individual experiments. *p<0.05, **p<0.01 vs control; #p<0.05, ##p<0.01 vs Tat-treated group.

Rat cortical neurons were prepared from prepared from 18-day-old fetuses of Sprague Dawley rats as previously described [39]. Pregnant rats were injected with an over-dose of pentobarbital (50 mg ? Rat) and the pups were removed and decapitated. Briefly, fetal rat brain cortices were harvested followed by mechanical trituration. Cells were then suspended in Neurobasal medium (Gibco) supplemented with 2 mM glutamine, 2% B-27 supplement and 1% antibiotic, and seeded at a density of 20,000 cells per well in 96-well plates pre-coated with poly-d-lysine and maintained at 37°C with 5% CO_2_. On day 3 of incubation, half the medium was changed and the cells were cultured for additional 3 days. Cell purity was determined by immunocytochemistry using anti-MAP-2 antibody (Chemicon, Temecula, CA) and were found to be >95% pure. All animal procedures were performed according to the protocols approved by the Institutional Animal Care and Use Committee of the University of Nebraska Medical Center.

### Cell Survival Assay

MTT (mitochondrial dehydrogenases [3(4,5-dimethylthiazol-2-yl)-2.5 diphenyltetrazolium bromide] was used to measure cell viability. 10 µl of 5 mg/ml MTT tetrazolium salt (Invitrogen, Grand Island, NY) was added to the media of treated SH-SY5Y cells or rat primary cortical neurons followed by incubation for 2–3 h at 37°C in 5% CO_2_ incubator. The medium was then aspirated and was replaced with 100 µl DMSO to dissolve the Formosan crystals in the cells. The amount of Formosan crystals was measured by absorbance using a Synergy MX plate reader (Biotek, Winooski, VT) at test and reference wavelengths of 570 and 630 nm respectively.

**Figure 3:**
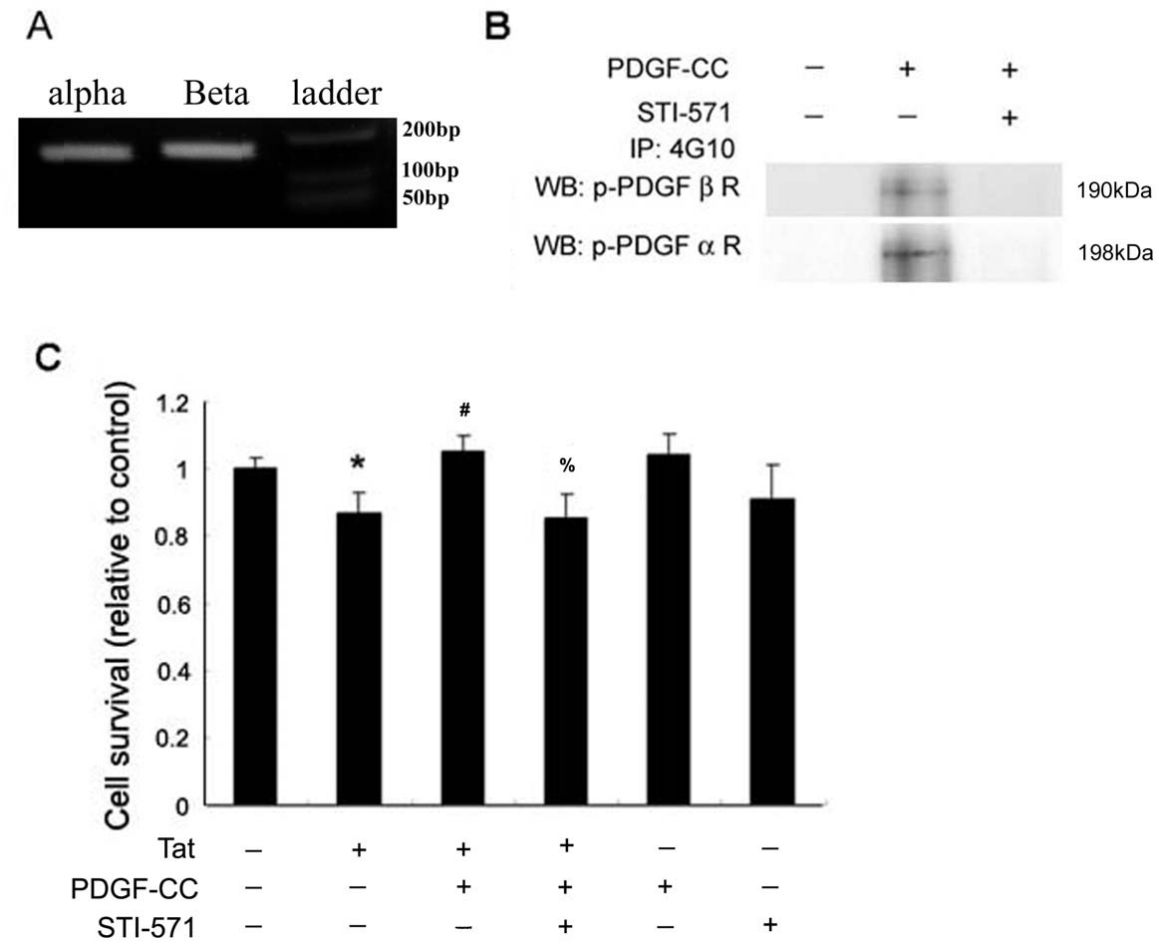
PDGF receptor is involved in PDGF-CC neuroprotection. (**A**) Expression of PDGF-αR and βR in SH-SY5Y cells by RT-PCR. (**B**) SH-SY5Y cells were exposed to PDGF-CC (50 ng/ml) for 30 min in presence or absence of tyrosine kinase inhibitor STI-571 (1 µM). Phosphorylation of PDGF-αR & βR was detected by co-immunoprecipitation. (**C**) Cell viability of SH-SY5Y cells exposed to PDGF-CC and/or Tat in presence or absence of tyrosine STI-571 was assessed by MTT assay. Figure is a representative of three independent experiments. All data in these figures are presented as mean ± SEM of three individual experiments. *p<0.05 vs control, #p<0.05 vs Tat-treated group, %p<0.05 vs PDGF-CC plus Tat-treated group.

### Cell Transfections and Adenovirus Infection

To confirm the roles of GSK3β, TRPC channels and Akt signaling in PDGF-CC-mediated neuroprotection, SH-SY5Y cells were transfected with either the wide-type or mutant GSK3β vector (WT or A9) (kind gift from Dr. J.Silvio Gutkind. National Institutes of health/NIDCR), TRPC 1, 5, 6 siRNA smart pool (Dharmacon, Chicago, IL) or infected with wild-type or dominant-interfering form of Akt adenovirus (kind gift from Dr. K. Walsh, Tufts University School of Medicine, Medford, MA). Lipofectamine 2000 (Invitrogen) was used to transfect cells with GSK3β vectors or TRPC channel siRNAs according to the manufacturer’s instructions. Briefly, SH-SY5Y cells were seeded in 96-well plate at a density of 2×10^4^ cells/well or in 24-well plate at 3×10^5^ cells/well. After 24 hrs medium was changed and replaced with serum free OPTI-MEM (Gibco). For transfection, 0.8 µg DNA vector or 100 nM siRNA in 100 µl of serum-free medium was mixed with 2 µl lipofectamine 2000, incubated at room temperature for 20 min and then added to the cell culture. Following gentle shaking and spinning at 1200 g for 5 min, transfected SH-SY5Y cells were cultured at 37°C for 24 h. For Akt adenovirus infection, SH-SY5Y cells were cultured as described above and then infected with either the wild type or dominant-interfering form of Akt adenovirus vector at a multiplicity of infection of 100 for 24 hrs.

**Figure 4:**
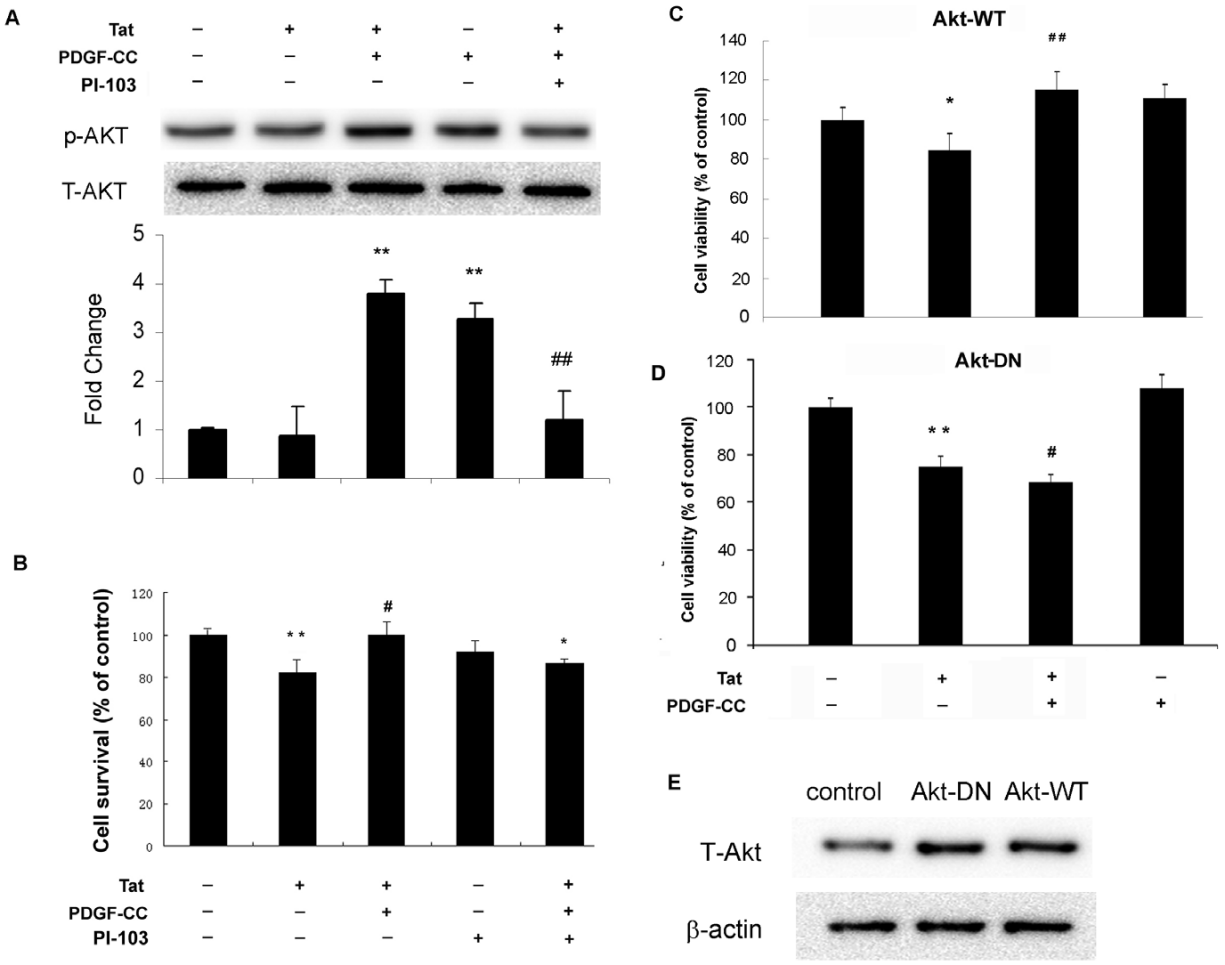
Akt is involved in PDGF-CC mediated neuronal protection. (**A**) SH-SY5Y cells were treated with PDGF-CC (50 ng/ml) and/or Tat (14 nM) in the absence or presence of PI3K/Akt inhibitor PI-103 (0.2 µM) followed by detection of Akt phosphorylation by western blot. (**B**) SH-SY5Y cells were treated as described for 24 h, and subjected to MTT assay. In panels C & D, SH-SY5Y cells were infected with either the wild type (**C**) or dominant-negative form of Akt adenovirus (**D**). (E) Equal expression levels of Akt in SH-SY5Y cells infection with Akt-DN or Akt-WT adenovirus. MTT assay was used to detect the cell viability of SH-SY5Y cells exposed to PDGF-CC and/or Tat. All data in the panels B, C & D are presented as mean ± SEM of three individual experiments. *P<0.05, **P<0.01 vs control; #p<0.05, ##p<0.01 vs Tat-treated group.

### Analysis of Neuronal Dendrites

For the measurement of changes in dendrite length induced by PDGF-CC and/or Tat, phase contrast images of SH-SY5Y cells were taken using the Carl Zeiss Axio Imager M2 fluorescence microscope (Carl Zeiss, Jena, Germany). The length of each fiber originating from each neuronal cell body and subsequent branches was measured, and a sum of total dendrite length for each neuron was calculated using Image J software (NIH). For each well, the pictures were taken from five areas randomly. Final results were expressed as a percentage of the control culture.

**Figure 5:**
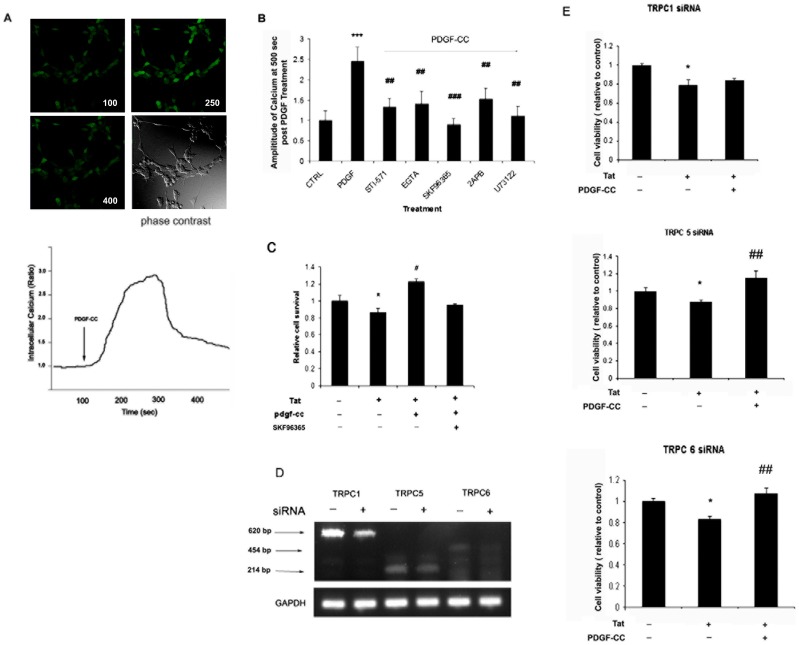
TRPC 1 channel is critical for PDGF-CC-mediated neuroprotection. (**A**) SH-SY5Y cells were labeled with Fluo-4 prior to treatment with PDGF-CC followed by recording of the fluorescence density representing the level of intracellular Ca^2+^. A representative change of fluorescence intensity is shown in the lower panel. The arrow indicates the time of addition of PDGF-CC. Number in upper panel shows the recording time. (**B**) Change in intracellular Ca^2+^ induced by PDGF-CC (50 ng/ml) in the presence of absence of pharmacological inhibitors (STI-571, 1 µM; EGTA, 2 mM; SKF96365, 20 µM; 2APB, 100 µM; U73122, 1 µM). All the data in these figures are presented as mean ± SEM of three individual experiments. ***p<0.001 vs control; ##p<0.01 vs PDGF-CC-treated group. (**C**) Cell viability in SH-SY5Y cells pretreated with SKF96365 (20 µM) by MTT assay. All the data in these figures are presented as mean ± SEM of three individual experiments. *p<0.05 vs control, #p<0.05 vs Tat-treated group. (**D**) SH-SY5Y cells were transfected with 100 nM siRNA targeting TRPC1, 5 or 6. After 24 h cells were lysed, mRNA was isolated and subjected to RT-PCR. Arrow indicates the RT-PCR product bands. (**E**) SH-SY5Y cells seeded in 96-well plate were transfected with TRPC 1, 5 or 6 siRNAs (100 nM) for 24 h later followed by treatment of cells with PDGF-CC and/or Tat. MTT assay was conducted to detect cell viability. All the data in these figures are presented as mean ± SEM of three individual experiments. *p<0.05 vs control; ##p<0.01 vs Tat-treated group.

### Western Blot Analysis

Treated cells were lysed using the Mammalian Cell Lysis kit (Sigma) containing protease and phosphatase inhibitors (Pierce, Rockford, IL) and the NE-PER Nuclear and Cytoplasmic Extraction Kit (Pierce) according to the manufacturer’s instructions. Equal amounts of the corresponding proteins were electrophoresed in a sodium dodecyl sulfate-polyacrylamide gel in reducing conditions followed by transfer to PVDF membrane. The blots were blocked with 5% nonfat dry milk in phosphate buffered saline. The western blots were then probed with antibodies recognizing the phosphorylated or total forms of PDGF-αR, PDGF-βR, Akt, and GSK3β, β-catenin, histone (Cell Signaling, Danvers, MA), PDGF-CC (Abcam) & β-actin (Sigma). All of the westerns were repeated at least three times.

**Figure 6:**
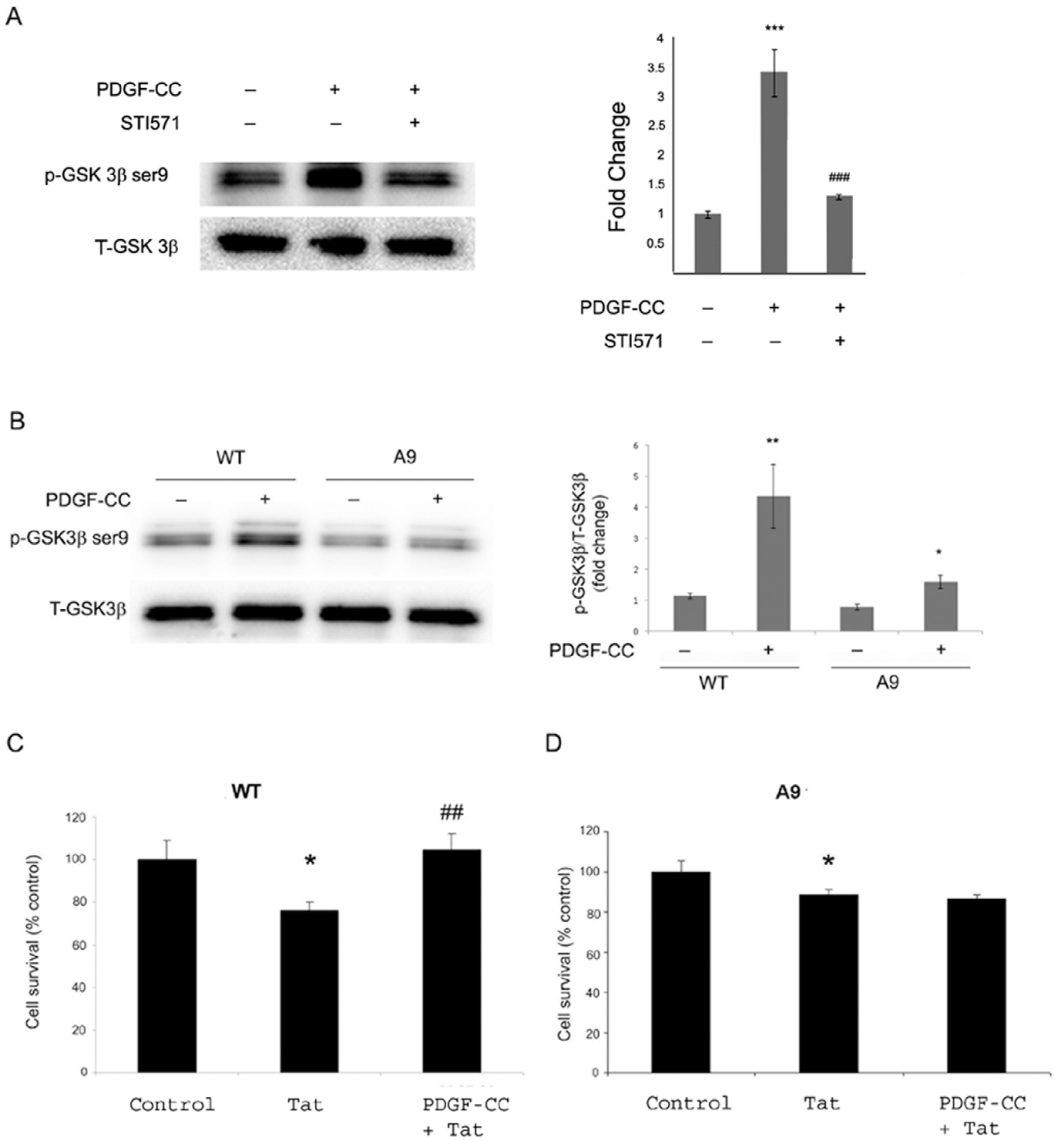
GSK3β plays the critical role in PDGF-CC-mediated protection. (**A**) Inactivation of GSK3β was monitored in SH-SY5Y cells treated with PDGF-CC in the presence or absence of STI-571 (1 µM). The data in this panel are presented as mean ± SEM of three individual experiments. ***p<0.001 vs control, ###p<0.001 vs PDGF-CC-treated group. (**B**) SH-SY5Y cells were transfected with either the wild type or A9 mutant GSK3β vector using lipofectamine 2000. After 24 h cells were treated with or without PDGF-CC, followed by cell lysis. Western blot was conducted to monitor alterations in phosphorylated GSK3β. Densitometric analysis of the WB is shown in the right panel. Figure is a representative of three independent experiments. All data in these figures are presented as mean ± SEM of three individual experiments. *p<0.05, **p<0.01 vs untreated cells. SH-SY5Y cells transfected with either the GSK3β WT or mutant A9 were incubated with PDGF-CC and/or Tat and assessed for cell viability using the MTT assay (**C** and **D**). All data are presented as mean ± SEM of three individual experiments. *p<0.05 vs control, ##p<0.01 vs Tat-treated group.

### Immunocytochemistry

For immunocytochemistry SH-SY5Y cells were plated on cover slips. After 24 hrs, cells were treated and fixed with 4% paraformaldehyde for 10 min at room temperature followed by permeabilization with 0.3% Triton X-100 in PBS. Cells were then incubated in a blocking buffer containing 5% BSA for 1 hr at room temperature followed by addition of mouse anti-MAP-2 (1∶10,000; Abcam) overnight at 4°C. This was followed by addition of the secondary Alexflour 488 goat anti-mouse IgG at a dilution of 1∶2000 for 2 h to detect expression of MAP-2. Cells were washed thrice in buffer and mounted with prolong Gold antifade reagent with DAPI (Invitrogen, Carlsbad, CA) onto slides. Slides were examined using the Carl Zeiss Axio Imager M2 fluorescence microscope (Carl Zeiss).

**Figure 7:**
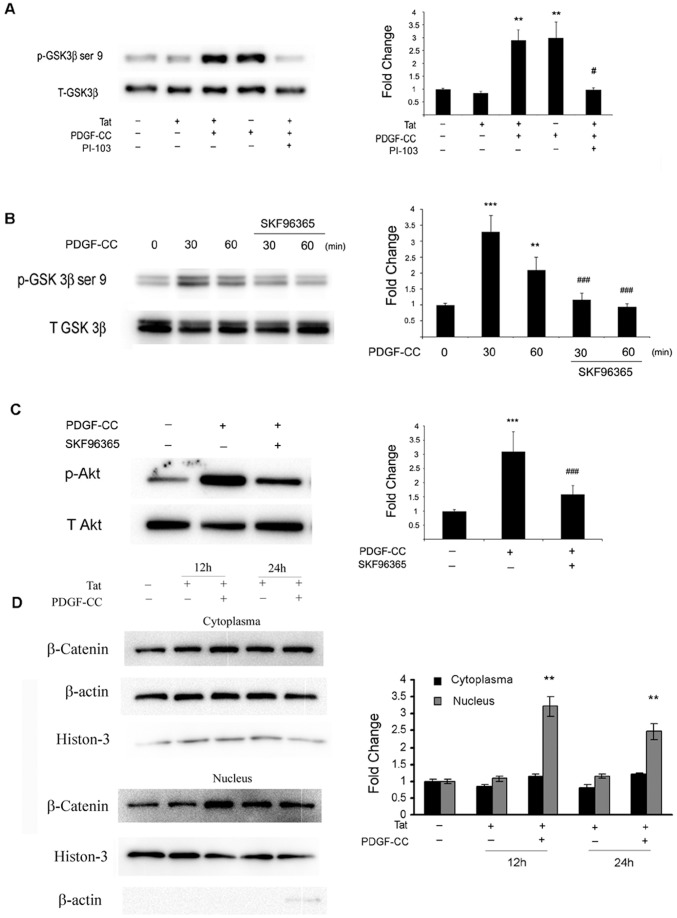
TRPC channel and Akt are involved in PDGF-CC mediated inactivation of GSK3β. (**A**) SH-SY5Y cells pretreated with or without the PI3K inhibitor PI-103 (0.2 µM), were exposed to PDGF-CC and/or Tat for 30 min. Cells were lysed, proteins were isolated and subject to WB to detect the phosphorylation of GSK3β. Data are presented as mean ± SEM of three individual experiments. **p<0.01 vs control, #p<0.05 vs PDGF-CC plus Tat-treated group. (**B**) WB demonstrating that blocking TRPC channel with SKF96365 resulted in loss of PDGF-CC mediated inactivation of GSK3β inactivation. Data were presented as mean ± SEM of three individual experiments. **p<0.01, ***p<0.001 vs control, ###p<0.001 vs PDGF-CC-treated group. (**C**) Treatment of cells with TRPC channel inhibitor SKF96365 (20 µM) resulted in mitigation of PDGF-CC-mediated activation of Akt. Data were presented as mean ± SEM of three individual experiments. ***p<0.001 vs control, ###p<0.05 vs PDGF-CC-treated group. (**D**) SH-SY5Y cells were treated with PDGF-CC (50 ng/ml) and/or Tat (14 nM) for the indicated times, and subjected to cytoplasm and nuclear protein extraction. WB analysis demonstrated PDGF-CC mediated accumulation of β catenin in nucleus. Figure is a representative of three independent experiments. **p<0.01 vs control.

### Co-immunoprecipitation

The procedure for immunoprecipitation was performed as described previously with slight modifications [40]. Briefly, SH-SY5Y cells treated with PDGF-CC and/or ST1571 were lysed in RIPA buffer (50 mM Tris, pH8.0, 150 mM NaCl, 0.1% SDS, 1.0% NP-40, and 0.5% sodium deoxycholate) containing proteinase and phosphatase inhibitors (Pierce). For each sample 200 µg of protein was used for co-immunoprecipitation. The sample protein was incubated with 2 µg of diluted anti-4G10 antibody (Millipore, Billerica, MA) overnight at 4°C followed by incubation with 30 µl protein A/G sepharose for 3 h at 4°C. The mixture was then centrifuged (at 600 g for 30 s) and the cell pellets were rinsed twice with RIPA, followed by boiling in 2X western blot loading buffer for 4 min. Following spinning (at 6000 g for 30 s) the supernatants were subjected to western blot as described above.

**Figure 8:**
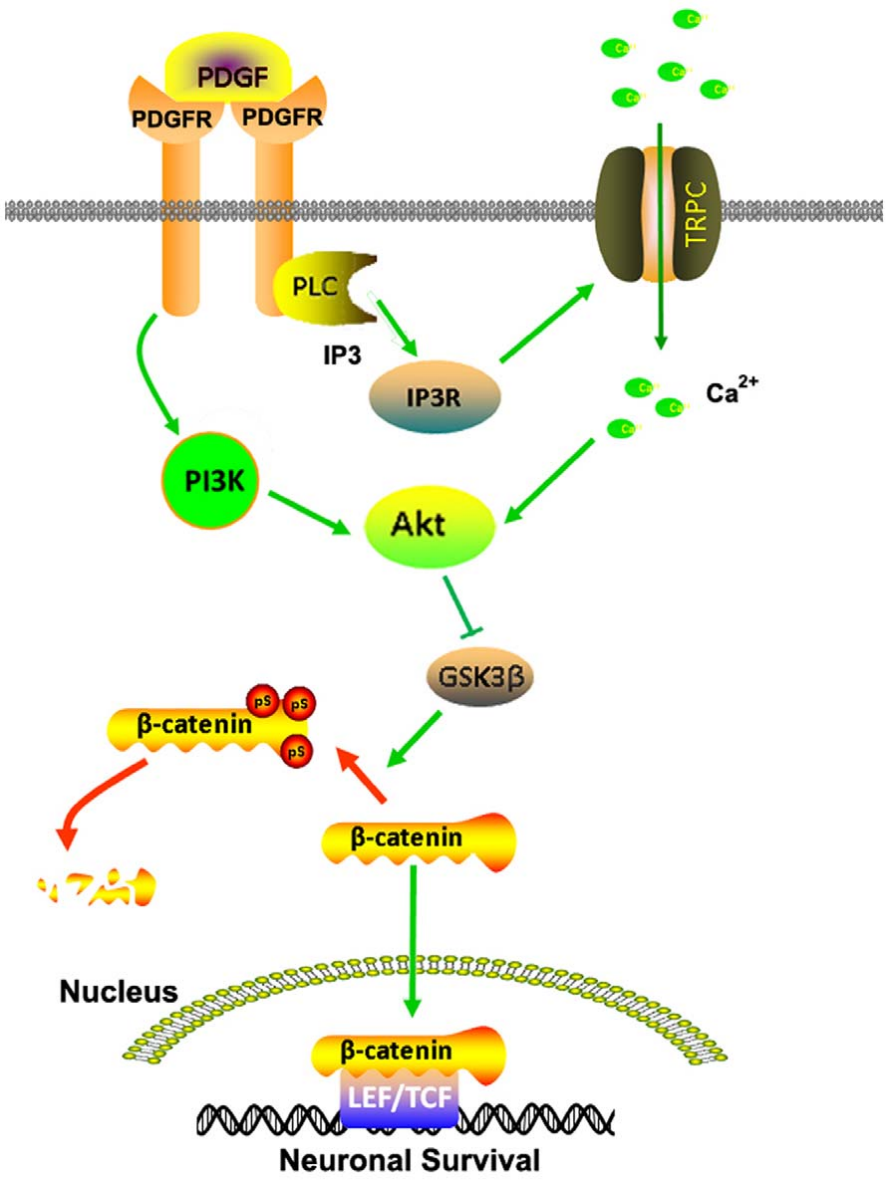
Schematic illustration demonstrating putative signaling pathways involved in PDGF-CC-mediated neuroprotection. PDGF-CC-mediated engagement of the PDGF receptor stimulates the PLC/PI3R pathway, which in turn, activates TRPC channels resulting in elevation of [Ca^2+^] transient. The elevation of [Ca^2+^] is required for PDGF induced PI3K/Akt pathway leading to inactivation of GSK3β signal. The inactivated GSK3β fails to induce the degradation of β-catenin resulting in accumulation of β-catenin in the cytoplasm, followed by its translocation into nucleus and subsequent induction of expression of genes associated with the cell survival.

### TUNEL Staining

SH-SY5Y cells were plated at a density of 1×10^5^ cells per well in a 24-well plate with cover slips for TUNEL staining. Following serum-starvation for 24 h and pretreatment with PDGF-CC and/or Tat for 16 h at 37°C, cells were washed with phosphate buffered saline (PBS) and fixed for 30 min with 4% paraformaldehyde at room temperature. The fixed cells were permeabilized with 1% Triton X100 for 30 min, followed by staining with TUNEL reaction mixture for 60 min, according to the manufacturer’s instructions (Roche, Palo Alto, CA). Coverslips were mounted using prolong Gold antifade reagent with DAPI (Invitrogen), followed by visualization of slides under the Carl Zeiss Axio Imager M2 fluorescence microscope (Carl Zeiss). Six to eight images per treatment group were analyzed. Blind image analysis of tunnel positive cells in various treatments was done using Image J software (NIH). Images from each slide were captured at 20× using Carl Zeiss Axio Imager M2 fluorescence microscope (Carl Zeiss). Threshold intensity for DAPI labeling was set to allow DAPI signals to be counted while eliminating false positive background staining. The number of DAPI-positive cells was then quantified for all images. Similarly, threshold intensity for TUNEL labeling was set to allow TUNEL-positive cells to be counted while eliminating false-positive background staining. After quantifying the number of TUNEL-positive cells for all the images, the percentage of TUNEL-positive cells to the total number of DAPI-positive cells was determined. The mean percentage (±SEM) of all images from each treatment group was reported.

### Reverse Transcription and Real-time PCR

In the present study, the expression of PDGF-CC, PDGFR-α or β and TRPC were detected by reverse transcription (RT) and real-time PCR as described previously [41]. Total RNA was extracted from cells using Trizol total RNA isolation reagent (Invitrogen) as detailed by the instructions from manufactures. For detection of expression of PDGF-CC and PDGFR, the quantitative polymerase chain reaction primers were purchased from SABiosciences. PCR condition was 5 min at 95°C of denature temp, 40 cycles of 30 s at 95°C, 30 s at 60°C of annealing temp, and 30 s at 72°C, and ended with 10 min at 72°C of extension temp.

The primer pair sequences for TRPC1, TRPC3, TRPC4, TRPC5, TRPC6, TRPC7 were the same as those published by Lu *et* al [42]. The PCR program was constituted of a denaturing step for 5 min at 95°C, 40 cycles of 30 s at 95°C, 30 s at 52°C and 30 s at 72°C, and a final extension for 10 min at 72°C. The primer sets for specific genes were based on a previous study [42].

### MAP-2 ELISA

The changes in expression of MAP-2 were determined by ELISA as previously described [22], [43]. Briefly, primary rat cortical neuron cultures were plated in 96-well plate at density of 2×10^4^ cells per well and treated as described above. Treated cells were fixed by 4% of Paraformaldehyde for 15 min at room temperature followed by 0.3% H_2_O_2_ in methanol to get rid of the endogenous peroxidase. The cells were then blocked with 5% normal goat serum in PBS and incubated for 1 h with 1∶10,000 MAP-2 antibody (Abcam), followed by incubation with anti-chicken biotinylated antibody at a dilution of 1∶10,000 (Vector Laboratories, Burlingame, CA) for 30 min. Antigen color development was done using Avidin/biotin system (Vector Laboratories) according to the manufacturer’s instructions, with the absorbance read at 450 nm using the Synergy MX plate reader (Biotek).

### Measurement of Free Intracellular Calcium

The changes in Ca^2+^ were monitored using fluo-4/AM (Molecular Probes, Eugene, OR) dissolved in dimethyl sulfoxide. Briefly, SH-SY5Y cells cultured in 35-mm culture dishes were rinsed twice with bath solution (140 mM NaCl, 5 mM KCl, 1 mM CaCl_2_, 0.5 mM MgCl_2_, 10 mM Glucose, 5.5 mM HEPES, pH7.4), followed by incubation in the same solution containing 5 µM Fluo-4/AM in 5% CO_2_, 95% O_2_ at 37°C for 40 minutes. Cells were then rinsed twice with the bath solution, mounted on a perfusion chamber, and scanned every five seconds using the Carl Zeiss ISM 510 confocal microscope (Carl Zeiss). Fluorochrome was excited at 488 nm and the emitted light was read at 515 nm. All analyses of Ca^2+^ were processed at a single-cell level. In order to normalize for variations in initial fluorescence values, the values of Ca^2+^ response were divided by the resting fluorescence value (calculated as the mean of at least three values prior to the application of PDGF-CC). All experiments were repeated at least three times with representative blots presented in the figures.

### Statistical Analysis

Statistical analysis was performed using one-way analysis of variance with a *post hoc* Student’s *t-*test. Results were judged statistically significant if p<0.05 by analysis of variance.

## Results

### Tat Down-regulates PDGF-C Chain Expression in Neurons

Because several neurodegenerative disorders are characterized by lack of trophic support [44], and based on our previous study that HIV associated protein gp120 was able to down-regulate the expression of trophic factors in neurons [41], we sought to explore whether HIV viral protein Tat could down regulate the expression of PDGF-CC. To test this, we used rat primary neurons and a neuroblastoma cell line, SH-SY5Y that was differentiated into neurons by retinoic acid treatment. Cells were exposed to 14 nM of Tat followed by collecting for RNA and protein extraction to monitor expression of PDGF-C chain by semiquantitative reverse transcriptase-polymerase chain reaction (RT-PCR) or Real-time quantitative PCR and western blot analyses, respectively. As shown in figure 1A, treatment of rat primary neurons or SH-SY5Y cells with Tat (14 nM) resulted in decreased expression of PDGF-C chain mRNA as early as 6 h post Tat challenge. This down-regulation of PDGF-C in was also confirmed by both RT-PCR (Fig. 1B) and western blot analyses (Fig. 1C).

### Protective Effect of PDGF-CC Against Tat-mediated Neurotoxicity

Since PDGF-CC is known to be expressed by healthy neurons [20] and has the neuroprotective potential [38], we hypothesized that pretreatment of neurons with PDGF-CC protein could protect against Tat-mediated toxicity. Treatment of SH-SY5Y or rat primary cortical neurons with Tat (14 nM) for 24 hrs followed by assessment of cell viability by MTT assay resulted in decreased cell viability (around 20%; p<0.01) as expected (Fig. 2A and B). These findings are in agreement with previous reports demonstrating neurotoxic role of Tat [10]. Interestingly, pretreatment of cells with PDGF-CC (50 ng/ml; concentration pre-determined based on the previous work [38]) for 30 min followed by exposure to Tat, resulted in increased cell viability. The exposure of preheated Tat that was included as a negative control, showed no toxicity to the neurons, as expected.

Since the exposure of neurons to HIV proteins leading to dendritic damage, is one of the mechanism(s) of neuronal degeneration [45], [46], we next sought to examine whether PDGF-CC could reverse Tat-mediated damage of neuronal dendrites in SH-SY5Y cells. As shown in figure 2C & 2D, exposure of neuronal cells to Tat for 5 days resulted in increased cell death compared to untreated cells with concomitant shortening of neurites by about 25%. Interestingly, pretreating the cells with PDGF-CC reversed Tat-mediated toxicity. These findings were consistent with previous reports demonstrating neurotoxic role of gp120 [41].

These findings were further validated by monitoring the expression of microtubule-associated protein-2 (MAP-2) using immunostaining and ELISA assays. As shown in panel 2E, Tat treatment of SH-SY5Y cells resulted in loss of MAP-2 immunoreactivity, an effect that was ameliorated by pretreatment of cells with PDGF-CC. Similar to immunostaining findings, semiquantitative MAP-2 ELISA assay also corroborated these findings (Fig. 2F).

It is well recognized that apoptosis plays a critical role in Tat-mediated neurotoxicity [47]–[49], and that neurotrophic growth factors in turn, can attenuate apoptosis induced by cerebral ischemia [50] and oxidative stress [51]. We therefore next sought to examine the ability of PDGF-CC to reverse the apoptosis induced by Tat. Using TUNEL assays, we demonstrated Tat-mediated induction of neuronal apoptosis and this was reversed in cells pretreated with PDGF-CC (Fig. 2G &H).

### PDGF Receptor is Involved in PDGF-CC Mediated Neuroprotection

Since binding of PDGF to its receptor mediates bioactivity of PDGF [41], [52], [53], we next sought to examine the role of PDGF receptor in the neuroprotection mediated by PDGF-CC. Expression of the two PDGF receptor isoforms PDGF-αR & -βR was first confirmed in SH-SY5Y cells by RT-PCR assay using the commercial Real-time primer set (SAB Bioscience, Santa Clarita, CA). As shown in figure 3A, there was abundant expression of both the receptors in these cells. Following treatment with PDGF-CC, both the receptors were rapidly phosphorylated, and this effect was abrogated in cells pretreated with the tyrosine kinase inhibitor STI-571 (1 µM) as evidenced by western blot (Fig. 3B).

Subsequently, the functional role of these receptors in PDGF-CC mediated neuroprotection was assessed by monitoring cell survival in the presence of Tat and STI-571 using the MTT assay. In SH-SY5Y cells pretreated with STI-571 PDGF-CC failed to reverse Tat-mediated decrease in cell survival (Fig. 3C), thereby underpinning the role of PDGF/PDGFR axis in neuroprotection against Tat.

### PI3K/Akt Pathway is Required for PDGF-CC Mediated Neuroprotection

PI3K/Akt signaling that is activated by G protein-coupled and tyrosine kinase receptors [54] plays a critical role in cell proliferation, survival and differentiation [55]–[57]. We wanted to gain insights into the role of this pathway in the protection conferred by PDGF-CC. To achieve this we resorted to both a pharmacological approach using inhibitors specific for PI3K and an adenovirus vector overexpressing either a wild type Akt (Akt-WT) or a dominant-interfering form of Akt (Akt-DN). As shown in figure 4, pretreatment of SY-SY5Y cells with the PI3K inhibitor PI-103 (0.2 µM) blocked PDGF-CC induced activation of AKT signal in SH-SY5Y cells (Fig. 4A) and consequently, completely abrogated PDGF-CC mediated neuroprotection against Tat cytotoxicity. For further validation of the role of Akt in PDGF-CC mediated neuroprotection, SH-SY5Y cells were infected with either the wild type (WT) or the dominant-interfering form (DN) of adenovirus Akt constructs followed by exposure to PDGF-CC and/or Tat. In cells transfected with WT Akt (Fig. 4C) PDGF-CC was able to reverse Tat-mediated neurotoxicity while in cells transfected with Akt DN (Fig. 4D), PDGF-CC failed to exert its protective effect, thereby underpinning the role of Akt in this process. Intriguingly, the decrease in cell survival in neurons treated with both Tat and PDGF-CC in the presence of Akt DN was greater than in cells treated with Tat alone.

### TRPC Channels Contribute to PDGF-CC Mediated Neuroprotection

Neurotrophic factors are known to exert their functions by regulating the intracellular lever of Ca^2+^ [58], [59], which play a key role in cell survival, proliferation and differentiation [60], [61]. We therefore rationalized that PDGF-CC could execute its neuroprotection by controlling the intracellular Ca^2+^ levels within the cell. TRPC is a member of TRP superfamily that functions as Ca^2+^ influx channels. We next sought to examine whether PDGF-CC was able to regulate Ca^2+^ influx via activation of TRPC channels. As shown in figure 5A & 5B, exposure of SH-SY5Y cells to PDGF-CC resulted in transient but significant up-regulation of intracellular Ca^2+^ levels. Intriguingly, this response was blocked in cells pretreated with STI-571, thereby underscoring the role of PDGF-CC in mediating Ca^2+^ influx. PDGF-CC mediated influx of Ca^2+^ was also abrogated by the Ca^2+^ chelator EGTA, thus suggesting the role of extracellular Ca^2+^ in this process.

Since TRPC channels are Ca^2+^ permeable cation channels, we next sought to explore whether TRPC channels played a role in PGDF-CC-mediated Ca^2+^ influx. Pretreatment of SH-SY5Y cells with the TRPC blocker SKF 96365, [62], [63], significantly reduced PDGF-CC mediated Ca^2+^ influx. To further confirm the relationship between PDGF signaling and TRPC channels, cells were pretreated with the inhibitors specific for IP3R (2ApB) or PLC (U73122), both of which are downstream mediators of PDGF-R signaling. Pre-treatment of cells with both the inhibitors resulted in in inhibition of Ca^2+^ elevation induced by PDGF-CC; thereby underpinning the role of PLC-IP3R pathway in PDGF-CC mediated signaling.

The next step was to examine the functional relevance of PDGF-CC mediated Ca^2+^influx in the neuroprotection mediated by this growth factor. As shown in figure 5C, inhibition of Ca^2+^ influx by pretreatment of cells with the TRPC blocker (SKF96365) resulted in failure of PDGF-CC in mediating neuroprotection against cytotoxicity induced by Tat. This finding highlights the role of TRPC channels in neuroprotection mediated by PDGF-CC.

Since TRPC comprises of a family of subtypes ranging from TRPC 1–7, we next investigated the specific TRPC subtype involved in this process. RT-PCR analysis revealed the presence of three TRPC channel subtypes (TRPC 1, 5 & 6) in SH-SY5Y cells (data not shown). To evaluate the role of each of these three channels in PDGF-CC-mediated neuroprotection SH-SY5Y cells were transfected with the respective siRNAs to knockdown the corresponding TRPC channel subtype (Fig. 5D). As shown in figure 5E, cells knocked down for TRPC1 but not for TRPC 5 or 6, exhibited a failure of PDGF-CC to mediate neuroprotection against Tat.

### GSK3β Plays the Critical Role in PDGF-CC Mediated Neuroprotection

GSK3β, which lies downstream of Akt, is an important signaling protein implicated in the translocation of several transcription factors that play a key role in the proliferation and differentiation of cells [64]. We sought to explore the role of GSK3β in PDGF-CC-mediated neuroprotection. As shown in figure 6A, treatment of SH-SY5Y cells with PDGF-CC resulted in inactivation of GSK3β as evidenced by its phosphorylation at serine 9. This inactivation by PDGF-CC was abolished in cells pretreated with the antagonist for tyrosine kinase receptor STI-571 (Fig. 6A) or in cells transfected with a serine mutant of GSK3β (Fig. 6B).

The next step was to investigate the functional role of GSK3β inactivation in PDGF-CC mediated neuroprotection using gain and loss of functions for GSK3β. In SH-SY5Y cells transfected with WT GSK3β (Fig. 6C), as expected, PDGF-CC induced neuroprotection against Tat, however, in cells transfected with the serine mutant of GSK3β (Fig. 6D), PDGF-CC failed to induce inactivation of GSK3β, thereby resulting in failure of the ability of PDGF-CC to induce neuroprotection.

### GSK3β is Critical for TRPC/Ca^2+^ Pathway-mediated Neuroprotection

Since GSK3β, TRPC and PI3K/Akt signaling pathways were all shown to be involved in PDGF-CC mediated neuroprotection, the next step was to investigate the relationship among these three key signaling molecules. In cells pretreated with the inhibitor of PI3 kinase, PDGF-CC failed to induce phosphorylation of GSK3β at serine 9 (Fig. 7A), thereby suggesting that PI3K/Akt pathway was up-stream of GSK3β. Similarly, by blocking PDGF-mediated Ca^2+^ influx using the TRPC blocker, SH-SY5Y cells failed to inactivate GSK3β (Fig. 7B), thereby suggesting that TRPC was upstream of GSK3β. The next step was to establish a link between TRPC and Akt. Cells were pretreated with the TRPC blocker and examined for expression of Akt. As shown in figure 7C inhibition of Ca^2+^ influx mediated by SKF96365 remarkably attenuated PDGF-CC mediated activation of Akt thereby underscoring the involvement of Ca^2+^ dependent Akt in this process.

β-catenin is well recognized as an important substrate of GSK3β signaling with a key role in neuronal survival and proliferation [65], [66]. In order to determine the contribution of β-catenin in PDGF-CC-mediated neuroprotection, SH-SY5Y cells treated with PDGF-CC and/or Tat for 12 h or 24 h were fractionated into cytoplasmic and nuclear compartments and subjected to western blot analysis. Intriguingly, treatment with of cells with PDGF-CC for either 12 h or 24 h resulted in nuclear accumulation of β-catenin (Fig. 7D).

## Discussion

HIV-associated CNS complication is characterized by varying levels of motor and behavioral dysfunctions leading to seizures, coma, and death within months [3] and is associated with neuropathology involving HIV-1 proteins and activation of proinflammatory cytokine circuits. Although the incidence of HAD has decreased considerably in the era of antiretroviral therapy (ART), its prevalence is on the rise with the emergence of a more subtle form of minor cognitive motor disorder [67]. HIVE, the pathologic correlate of HAD reveals a broad spectrum of pathological changes, including multifocal and subacute encephalitis, focal accumulation of macrophages and multinucleated giant cells, widespread reactive astrogliosis, cerebral cortical atrophy, loss of specific neuronal subpopulations, and diffuse white matter pallor [4]–[6], [68]. Neuronal dysfunction/loss associated with HAD is often exemplified by loss of synapses, shortening of neuritis, and appearance of dendritic abnormalities [2]. Interestingly, unlike most other viral encephalopathies, neuronal loss associated with HIVE is not due to direct viral infection of the neurons. One broad explanation frequently advocated to explain this indirect killing of neurons is attributed to the toxicity mediated by the viral proteins, including gp120, that are released from virus infected cells in the CNS [69]–[74].

There exists a sensitive balance between neuroprotective and neurotoxic factors in the CNS exposed to insults. Neurotrophic growth factors such as BDNF, FGF and NGF [16]–[19] are known to play a key role in CNS homeostasis. In our previous study, we had reported that in macaque brains with Simian-HIV encephalitis there was down regulation of the neurotropic factor – the B Chain form of PDGF, and that it was also able to protect primary neurons against toxicity mediated by HIV env protein gp120 as well as Tat [41]. In the present study, we examined the neuroprotection against yet another member of this family of growth factors PDGF-CC, which in recent year has been shown to protect neurons from axotomy or ischemia-induced neuronal death [75] but not in the context of HIV/HIV protein toxicity.

Herein we show that rat primary neurons and SH-SY5Y cells exposed to Tat demonstrated decreased expression of the neurotrophic protein PDGF-CC. Concomitantly, Tat challenge led to neuronal injury as evident by increased expression of TUNEL positive cells, loss of cell viability, shortening of neurite length and decrease of MAP-2 expression. Intriguingly, pretreatment of neuronal cells with PDGF-CC reversed Tat-mediated neuronal injury. This was in agreement with the neurorprotective role of trophic factors such as FGF and BDNF against HIV protein toxicity [76], [77].

Accumulated evidence implies the involvement of PDGF-mediated PI3K/Akt signaling pathway as a key player in cell survival [78], [79]. This pathway plays a vital role not only in neurons but also in various other cell types [80], [81]. PI3K/Akt pathway is a central node in cell signaling downstream of growth factors, cytokines and other cellular stimuli [82]. In agreement with the earlier published reports [38], we also observed that PDGF-CC mediated neuroprotection against toxicity induced by Tat involved PDGF-receptor dependent PI3K/Akt signaling. Further validation of Akt signaling was confirmed using gain and loss of function in cells infected with either the wild type or dominant-interfering form of Akt adenovirus. Intriguingly, the decrease in cell survival in neurons treated with both Tat and PDGF-CC in the presence of Akt DN was greater than in cells treated with Tat alone. This could be attributed to a signaling pathway induced by PDGF that is distinct from Akt but that could perhaps be involved in cell toxicity in the absence of Akt.

In addition to Akt pathway, Ca^2+^ signaling has also been implicated as a major player in regulating cell survival and differentiation [60], [61]. Ca^2+^ overloading is thought to regulate many key steps in both cell survival and death mechanisms [83], [84]. In recent years TRPC-mediated Ca^2+^ influx has been suggested to play a role in neuronal survival [85]. A novel finding of our study was that PDGF-CC triggered Ca^2+^ transients by controlling the activity of TRPC channels, which consequently, regulated cell survival. Mammalian TRPC channel family consists of seven members that appear to function as receptor-operated channels [86]. With the exception of TRPC2, these channels are widely distributed in the mammalian brain. In our studies, three subtypes of TRPC channels (1, 5 & 6) were found to be expressed in SH-SY5Y cells. Using pharmacological inhibitor, SKF96365 we further demonstrated the role of Ca^2+^ channel in PDGF-CC-mediated neuroprotection. However, owing to the inherent non-specificity of the pharmacological inhibitor SKF96365, we sought to use an additional knock down approach using siRNA for the respective TRPC subtypes. Using such an approach we demonstrated that loss of TRPC1 expression, but not TRPC 5 or 6, resulted in abrogation of PDGF-CC-mediated neuroprotection. These findings are in agreement with previously published reports [31], [32], wherein both IP3R and PLC were shown to be mediating the interaction of PDGFR and TRPC channel.

Activation of the PI3K/Akt singling pathway is known to result in the phosphorylation of the downstream mediator –GSK3β, a physiologically relevant principle regulatory target of the Akt pathway [87]. GSK3β had been initially identified as a key regulator of insulin-dependent glycogen synthesis and is known to function in diverse cellular process including proliferation, differentiation, motility and survival [88], [89]. Following GSK3β inactivation, there is accumulation of its substrate - β-catenin. Reciprocally, in the presence of active GSK3β, there is degradation of β-catenin. Following translocation into the nucleus, β-catenin can bind with the transcription factors, T-cell factor/lymphoid enhancer binding factor (TCF/LEF), resulting in the promotion of cell survival [90]. TCF/LEF is a group of down-stream transcription factors of the Wnt/β-catenin signal pathway that is implicated in survival of neuronal cells [91]–[93], by controlling several cell survival-related genes, including neurotension receptor 1 gene [94], calcium/calmodulin-dependent protein kinase IV (CamK4) [95]. In the present study, we demonstrated PDGF-CC mediated activation of Akt followed by inactivation of GSK3β. Functional role of GSK3β inactivation in PDGF-CC-mediated neuroprotection was demonstrated using the gain and loss of function approach in cells transfected with either the wild type or the mutant GSK3β construct respectively. In concordance with the previously published findings on the role of β-catenin in cell survival, we also demonstrated increased accumulation of β-catenin following PDGF-CC exposure of cells.

The present study unravels the novel role of PDGF-CC as a neurotrophic factors against toxicity induced by HIV protein Tat. The mechanism of PDGF-CC-mediated neuroprotection, involved PDGF-R dependent activation of Akt signaling via the PI3K and TRPC pathways with downstream intersection and inactivation of GSK3β, thereby highlighting the critical role of GSK3β in neuroprotection (Fig. 8). These findings could pave way for development of possible therapeutic options for the treatment of neurodegeneration that is associated with HIV-infection.
